# Dynamic Adhesive Behavior and Biofilm Formation of *Staphylococcus aureus* on Polylactic Acid Surfaces in Diabetic Environments

**DOI:** 10.3390/ma17133349

**Published:** 2024-07-06

**Authors:** María Fernández-Grajera, Miguel A. Pacha-Olivenza, María Coronada Fernández-Calderón, María Luisa González-Martín, Amparo M. Gallardo-Moreno

**Affiliations:** 1 Networking Research Center on Bioengineering, Biomaterials and Nanomedicine (CIBER-BBN), 06006 Badajoz, Spain; mariafg@unex.es (M.F.-G.); mpacoli@unex.es (M.A.P.-O.); koferca@unex.es (M.C.F.-C.); amparogm@unex.es (A.M.G.-M.); 2 University Institute of Extremadura Sanity Research (INUBE), 06006 Badajoz, Spain; 3 Department of Biomedical Science, University of Extremadura, 06006 Badajoz, Spain; 4 Department of Applied Physics, University of Extremadura, 06006 Badajoz, Spain

**Keywords:** polylactic acid, diabetes, infection, glucose, ketone bodies, *Staphylococci*

## Abstract

Interest in biodegradable implants has focused attention on the resorbable polymer polylactic acid. However, the risk of these materials promoting infection, especially in patients with existing pathologies, needs to be monitored. The enrichment of a bacterial adhesion medium with compounds that are associated with human pathologies can help in understanding how these components affect the development of infectious processes. Specifically, this work evaluates the influence of glucose and ketone bodies (in a diabetic context) on the adhesion dynamics of *S. aureus* to the biomaterial polylactic acid, employing different approaches and discussing the results based on the physical properties of the bacterial surface and its metabolic activity. The combination of ketoacidosis and hyperglycemia (GK2) appears to be the worst scenario: this system promotes a state of continuous bacterial colonization over time, suppressing the stationary phase of adhesion and strengthening the attachment of bacteria to the surface. In addition, these supplements cause a significant increase in the metabolic activity of the bacteria. Compared to non-enriched media, biofilm formation doubles under ketoacidosis conditions, while in the planktonic state, it is glucose that triggers metabolic activity, which is practically suppressed when only ketone components are present. Both information must be complementary to understand what can happen in a real system, where planktonic bacteria are the ones that initially colonize a surface, and, subsequently, these attached bacteria end up forming a biofilm. This information highlights the need for good monitoring of diabetic patients, especially if they use an implanted device made of PLA.

## 1. Introduction

Diabetes is a chronic pathology that is increasing in the world population [1,2], in which patients suffer from alterations in blood glucose levels and ketone bodies. For example, high concentrations of glucose (hyperglycemia) and ketone bodies (ketoacidosis) negatively affect the immune system, hindering healing [3,4,5,6] or angiogenesis, causing impaired vascularization [6,7,8,9]. Moreover, this disease increases the probability of developing infections with potentially severe consequences. Specifically, in infections associated with implants, diabetes promotes complications such as osteomyelitis, causing a prosthesis to loosen, leading to its removal. As early as 2018, the work of Carey et al. [10] reported a statistical study demonstrating the connection between diabetes and infection, finding the highest incidence ratios for bone and joint infections and sepsis in diabetic scenarios.

However, little knowledge has been provided on how these diabetic environments could affect the microorganisms involved in infections. In the case of implant-associated infections, glucose and ketone bodies can cause changes in the adhesive properties of pathogenic bacteria [11]. A number of reviewed papers comment that the “increased” pathogenicity of diabetic-associated infections is not due to increased resistance of the bacteria themselves but to diabetes-associated immune deficiencies and circulatory deficiencies in the vascular system [12]. Diabetic patients have an altered function of polymorphonuclear cells and impaired phagocytosis, chemotaxis, and bactericidal activity, which are more evident in the presence of high hyperglycemia [13,14,15].

The process by which bacteria colonize a surface consists of two phases. In the first phase, adhesion to surfaces is governed by physicochemical interactions arising from the physical and chemical properties of bacteria and the host surface. Subsequently, microorganisms consolidate their attachment to the surfaces through biochemical interactions and bacterial surface structures. Following this initial phase, bacterial proliferation occurs and forms what is known as a biofilm [16,17,18]. These structures are bacterial communities attached to the surface with an extracellular matrix that provides protection against the host immune system and antimicrobials, making them highly resistant to eradication [19]. The analysis of this process can be difficult because many factors are involved, such as the environment, bacterial surface properties, or even metabolic states.

Different materials are employed for the fabrication of implants, but there is increasing interest in the use of bioresorbable materials. Among them, degradable polymers such as polylactic acid are the focus for such medical purposes [20,21,22,23]. Its versatility, improved by the addition of other compounds or by mixing it with other polymers, allows for the adjustment of its properties to meet the needs of different applications [24,25,26,27,28,29,30,31]. PLA is used in systems of microparticles for drug delivery and as support in scaffolds, helping in tissue regeneration for wound healing or for proper tendon healing. PLA has even demonstrated success in orthopedic devices in animal models: in the market there are screws, pins, and plates fabricated with PLA. Furthermore, its capability of being used in 3D printing enhances its present and future interest for more personalized medical care [32]. Diabetic patients can benefit from using PLA devices, such as sutures, plates, or even scaffolds for wound healing. Nevertheless, despite such valuable characteristics, PLA is not free from being susceptible to bacterial colonization.

One type of bacteria found in a large number of implant-associated infections with a high virulence is *Staphylococcus aureus* [33]. Glucose is a source of energy for *S. aureus*, which is converted into pyruvate and then produces ATP via the metabolic pathway of glycolysis. Intermediate products in this pathway, such as pentose phosphate, are important for nucleotide biosynthesis and defense against reactive oxygen species (ROS). These pathways can be affected by hyperglycemic conditions and then on the virulence factors of *S. aureus* [34]. On the other hand, ketone bodies are also an energy source through the action of ketogenic and ketolytic enzymes, which are able to synthesize and break down poly-3-hydroxybutyrate. D-3-hydroxybutyrate dehydrogenase would lead to an increase in the NADH/NAD+ ratio and citrate, related to the inhibition of bacterial growth [35]. Also, bacterial metabolism is affected under hyperglycemic and ketoacidosis conditions, and the adhesion capacity of bacteria is also altered because glucose and ketone body molecules can adhere to the bacteria, modifying the surface charge or the pH conditions in the surrounding media [36]. Better knowledge of how this bacterium responds to adhesive processes in environments supplemented with glucose or ketone bodies would help in understanding the extra complications of infectious processes in diabetic patients [5,37,38,39,40,41]. There are different models to study the microbial colonization processes in vitro. Specifically, in dynamic adhesion models, the microorganisms are allowed to be in contact with the biomaterial surface at a controlled and constant flow rate. This allows them to work in conditions closer to in vivo because it is unusual to find a strictly static environment inside the human body. This type of test also allows for the evaluation of the progression of the adhesion process over time and can be of particular importance in biodegradable materials, where their surface can be more significantly modified during contact with laminar flow and, as a consequence, can alter bacterial adhesion.

This study aims to provide better knowledge of the mechanisms underlying bacterial adhesion and biofilm formation in diabetic environments of *S. aureus* strains during the colonizing of a PLA surface, focusing on the interactions between the bacterial cells and the biomaterial, as well as the biological activity of the microorganisms. Understanding these mechanisms is crucial for developing strategies to prevent and manage implant-associated infections in diabetic patients, thus improving future clinical outcomes.

## 2. Material and Methods

### 2.1. Sample Preparation

Poly(lactic acid) was supplied in particulate form. This amorphous polymer was purchased from PURASORB© (Corbion, Amsterdam, the Netherlands). The samples were made with the solvent-casting protocol by Luque-Agudo et al. [42]. The PLA particles were dissolved in chloroform (20% *w*/*v*) using a rotary stirrer (JP Selecta, Barcelona, Spain) at room temperature. Then, 0.8 µL of the PLA’s solution was deposited on glass disks (25 mm diameter and 2 mm thickness) (Garvaglass S.L.L., Santa Perpetua de Mogoda, Barcelona, Spain) and dried at room temperature for 24 h. Afterwards, samples were dried in an oven at 70 °C for 24 h in order to completely remove any remaining solvent. Prior to use, the glass disks were cleaned by immersing them in chromic acid for 15 min and then rinsed with deionized water. After the cleaning process, they were stored in desiccators until use. In a previous study [43], physical surface properties of the film were analyzed, yielding a surface roughness of 0.36 ± 0.02 nm, without any apparent porosity, whose size could affect bacterial retention as observed from AFM analysis.

Physical Characterization of PLA Surfaces

The hydrophobicity of the PLA surfaces was obtained by measuring the water contact angle, using a Krüss goniometer (Krüss, Hamburg, Germany), as previously reported [43,44]. Briefly, 5 µL drops of deionized water were placed at different positions on the surface of the samples, and, after reaching the equilibrium state after 15 s, they were photographed and analyzed with Drop Shape Analyzer software (Version 1.12.2.06901, Krüss GmbH, Hamburg, Germany). Prior to measurements, the samples were immersed in each one of the diabetic environments (as specified in the next section), and adsorption was allowed for at least 2 h. After this time, the samples were left to air dry for 24 h, until completely dry, and then the water contact angle was measured.

The zeta potential of the films was obtained from streaming current measurements using an Electrokinetic Analyser (EKA, Anton Paar KG, Graz, Austria), and operation was similar to that previously described [45]. A commercial clamping cell (Anton Paar) was used with a PMMA sample (given by the manufacturer) facing the samples to be measured. The system was operated in an alternating pressure ramp from 0 to 300 mbar. Each measurement was the average of 8 cycles using 10^−3^ M KCl electrolyte solution in Millipore water, with or without supplementation of diabetic compounds, as described in the next section. The zeta potential was calculated using the Helmholtz–Smoluchowski equation:

(1) $$\zeta_{cell}=\frac{\eta }{\varepsilon }\frac{L}{S}\frac{dI_{str}}{dp}$$

where *ε* and *η* are the permittivity and viscosity of the electrolyte, respectively; *L* and *S* are the length and cross-section of the electrokinetic channel; and *p* and *I_str_* are the pressure and streaming current difference between both ends of the channel, respectively.

Due to the asymmetry of the cell used, the following equation has been applied to obtain the zeta potential of each sample:

(2) $$\zeta_{cell}=\frac{1}{2}\zeta_{PMMA}+\frac{1}{2}\zeta_{PLA}\to \zeta_{PLA}=2\zeta_{cell}-\zeta_{PMMA}$$

where *ζ_PMMA_* has been calculated with Equation (1) using the reference PMMA sample for each experimental condition.

### 2.2. Bacterial Strain and Growth Media

Gram-positive *Staphylococcus aureus ATCC 29213*, obtained from The American Type Culture Collection (ATCC, Manassas, VA, USA), was used in this study and was stored at –80 °C in porous beds (Microbank Pro-Lab Diagnostics, Austin, TX, USA). From the frozen stock, blood agar plates (OXOID Ltd., Basingstoke, Hampshire, UK) were inoculated and incubated at 37 °C. A preculture was made from a bacterial colony taken from these plates, and it was incubated at 37 °C for 9 h with Trypticase Soy Broth (TSB) (OXOID Ltd., Basingstoke, Hampshire, UK). After that, 50 mL of glucose-free TSB (Sigma-Aldrich, St. Louis, MO, USA), with or without supplements, was inoculated with 25 µL of this preculture and incubated for about 14 h at 37 °C to perform the adhesion experiments. The incubation time almost matches with the end of the exponential phase of bacterial growth for each experimental condition [36].

The diabetic environment model was simulated with different concentrations of glucose and ketone bodies present in diabetic patients and compared to those of healthy individuals. The concentrations selected were based on the literature and our previous studies [46,47,48,49,50,51]. Briefly, fasting blood glucose values show that <1 g/L is non-diabetic, and >1.26 g/L is diabetic or hyperglycemic. In relation to ketone bodies, the literature shows that normal ketone body levels are ≤1 mmol/L, and ketoacidosis levels are ≥3 mmol/L.

Glucose (Sigma-Aldrich, St. Louis, MO, USA) was added into the TSB medium at 0.9 g/L (normal level, G1) or 1.9 g/L (hyperglycemic level, G2). Ketone bodies were simulated to match real body conditions by mixing acetone (Panreac Chemistry SLU, Barcelona, Spain), methyl acetoacetate (ACE) (Sigma-Aldrich, St. Louis, MO, USA), and hydroxybutyric acid (HA) (Sigma-Aldrich, St. Louis, MO, USA) at 1:6 for HA:ACE and 2% acetone [48]. Considering these proportions, experiments were conducted at 1 mmol/L (normal level, K1) or 9 mmol/L (ketoacidosis level, K2) of ketone components. Combined glucose + ketone body conditions were obtained by mixing G1 + K1 (GK1) and G2 + K2 (GK2). Solutions with neither glucose nor ketone bodies were used as the control.

Further experiments were carried out by adding the diabetic components in the growth media and in the bacterial adhesion suspensions.

### 2.3. Bacterial Adhesion

#### 2.3.1. Experimental Parameters

Dynamic adhesion tests were made in a laminar flow chamber with parallel flat plates, as described in the work of Sjollema et al. [52]. Due to its design, it was possible to directly observe the adhesion process using an inverted metallographic microscope GX51 (Olympus, Barcelona, Spain) with a 20× objective and an attached camera connected to a computer.

For this experiment, after bacterial growth, bacteria were centrifuged and washed three times in phosphate buffer saline (PBS) without and with diabetic supplements (the same as those contained in the growth media). Then, they were resuspended in 250 mL of fresh PBS (with and without supplements) at a final concentration of 3 × 10^8^ CFU/mL (62% of transmittance at 492 nm) using a horizontal light spectrophotometer (Helios epsilon model, Thermo Spectronic, Thermo Fisher Scientific Inc., Cambridge, UK). First, the adhesion chamber was pre-conditioned with PBS for 15 min at a rate of 2 mL/min using a peristaltic pump. Then, the PBS was replaced by the bacterial suspension, which was maintained at 37 °C with the help of a thermostatic water bath.

Adhesion quantification was performed by analyzing the captured images at specific interval times for 5 h. During the first 10 min, images were captured every 30 s and in the following 10 min, every 60 s. From the 20th minute onward, images were taken every 5 min until completing 3 h of the experiment, and from this time until the end of the experiment, images were captured every 15 min. To quantify the bacteria adhered to the surface, the NIS-Elements BR 4.10 program (Nikon Instruments Inc., Melville, NY, USA) was used. To evaluate the bacterial retention capacity of the surface, at the end of the 5 h adhesion period, an air bubble was passed through the tubes and the flow chamber, and the detachment percentage was obtained (*D*).

All measurements were performed in triplicate with independent cultures. The results were expressed with their mean values and standard deviations from the mean.

#### 2.3.2. Data Analysis

Following the strategies proposed by Busscher and van der Mei [53] for the study of dynamic adhesion systems, two different methods of analysis have been used to interpret the information provided from the adhesion data.

The first analysis was based on fitting all data coming from the bacterial adhesion curve. This analysis considers the progressive filling of the surface, which in turn is a function of the coverage state over time. Systems like that have an exponential behavior defined by

(3) nt=n3001−e−tA

where $n_{300}$ is the “stationary” final number of bacteria attached at the end of the experimental time, *t* is the time, and *A* is the characteristic adhesion time, which is specific for each experimental condition and refers to the time required for *n*(*t*) = $n_{300}$ if the initial surface covering rate were constant.

The second approach linearizes the first section of the experimental curve $n\left(t\right)$ and analyzes the initial bacteria-surface affinity through the initial adhesion rate ($j_{0}$), which is the slope of this linear fit. Also, it takes into account the number of bacteria attached at the end of the experiment ($n_{e}$), which can be that belonging to the stationary state, if reached. The number of bacteria detached from the material surface after exposure to a liquid–air interphase was expressed as a detachment percentage (*D*).

### 2.4. Bacterial Metabolic State

In order to obtain insight into the possible causes of the adhesive behavior of bacteria when they are subjected to diabetic environments, one of the tests carried out was the study of the metabolic state of the bacteria prior to and after being in contact with the diabetic supplements for a time equal to the adhesion time.

For this purpose, the relative change in ATP produced by bacteria suspended in supplemented media compared to a control, that is, bacteria suspended in non-supplemented media, was examined. The bacteria, once grown in the media enriched or non-enriched with the diabetic components, were suspended in PBS enriched with the diabetic supplements to a concentration of 62% transmittance and kept at 37 °C for 300 min, which are the same temperature and time conditions of adhesion process. After this time, 600 µL were extracted from each system, and aliquots of 100 µL were deposited in six wells of a microtiter plate. Next, 100 µL of BacTiter-Glo™ Microbial Cell Viability Assay was added to each well, and after 5 min of incubation in the dark, the emitted luminescence was measured. The amount of relative light units (RLU) is directly associated with the metabolic state of the bacteria. Results will be presented as the percentage of RLU with respect to the RLU measurement for the control system.

### 2.5. Biofilm Formation

The protocol detailed in previous studies [54,55] was followed to analyze the formation of biofilms on PLA. Specifically, after growth, bacterial suspension was adjusted at 82% of transmittance at 492 nm using a horizontal light spectrophotometer (Helios epsilon model, Thermo Spectronic, Thermo Fisher Scientific Inc., Cambridge, UK) and then was diluted 1/100 to obtain a concentration of approximately 10^6^ CFU/mL. The PLA samples were fixed to a flat support with double-sided sticky tape, and then, 1 mL of the bacterial suspension, with the diabetic supplements, was kept in contact with the samples, with the help of silicone adhesion chambers, for 24 h at 37 °C under orbital shaking. After the incubation time, the supernatant was removed from each sample and carefully washed twice with TSB to remove unattached bacteria. The estimation of the viable biofilm-forming bacteria was performed by the comparison of their ATP production with respect to the control biofilm, that is, the biofilm grown with non-enriched media, using BacTiter-Glo™ Microbial Cell Viability Assay. After 5 min in darkness and light agitation, the supernatant was transferred to a 96-well white polystyrene flat-bottom microtiter plate (Greiner bioone, Frickenhausen, Germany), and the emitted light was quantified with a fluorescence microplate reader (FLx800; Bio-Tek Instruments, Inc., Winooski, VT, USA). Results will be presented as the percentage of RLU with respect to the RLU measurement for the control system. Experiments were performed in triplicate and with independent cultures to confirm reproducibility.

### 2.6. Statistical Analysis

All measurements were performed at least in triplicate with independent cultures. Results were expressed as mean values and standard deviations. Statistical analyses were performed with the R statistical software package (The R Foundation for Statistical Computing, www.r-project.org-R Core Team, 2014). Data were analyzed by one-way analysis of variance (ANOVA) with a Tukey’s post-hoc test. Statistical significance was considered at *p* < 0.05.

## 3. Results and Discussion

### 3.1. Bacterial Adhesion under Dynamic Conditions on Polylactic Acid Films in an Enriched Environment with Diabetic Conditions

The study of bacterial adhesion in diabetic environments has shown that all the systems had good reproducibility. The dynamic adhesion plots present the number of bacteria adhering per unit area (data in the order of 10^6^ bacteria/cm^2^) against the adhesion time. The “filling” process of the surface was analyzed with two approximations based on dynamic adhesion models.

**Figure 1:**
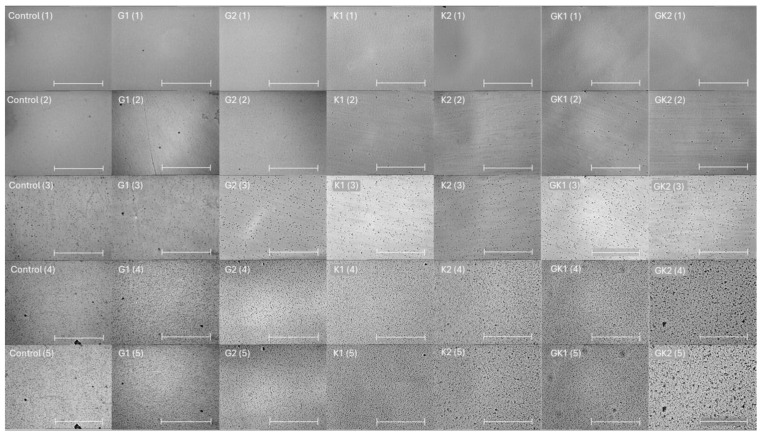
Microscopy images taken at 0 min (1), 0.5 min (2), 10 min (3), 180 min (4), and 300 min (5) during the dynamic bacterial adhesion experiments of *S. aureus* to the PLA surface into each experimental condition. Scale bar in the bottom right corner represents 36 µm.

Figure 1 shows a representative image of the bacterial adhesion process to the PLA surface. Each column is associated to one type of bacterial environment, and each row shows a specific adhesion time. The number of total bacteria per unit area is plotted and analyzed in the following figures.

#### 3.1.1. First Analysis Strategy: Exponential Approximation

**Figure 2:**
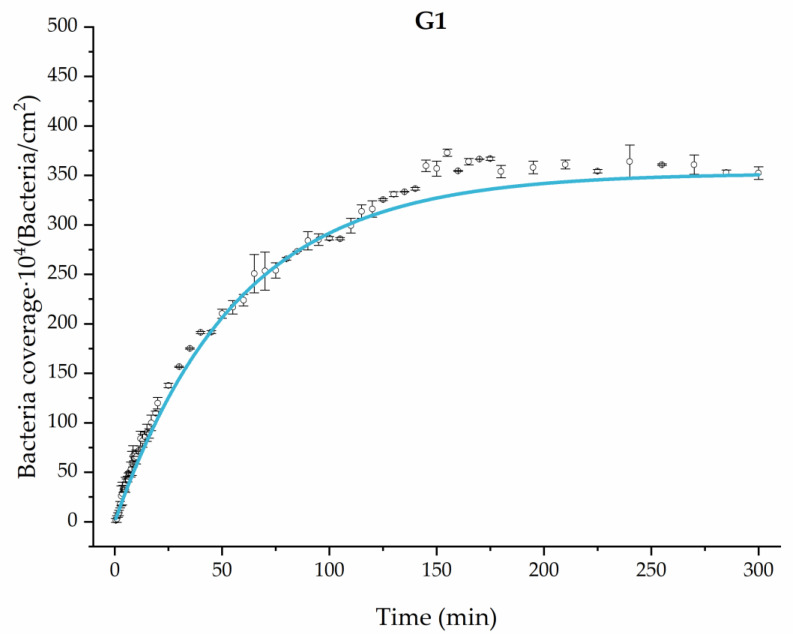
Example of the exponential fit of the system enriched with 0.9 g/L glucose on growth and adhesion (G1). Images taken every 30 s during the first 10 min. Images taken every 60 s during the next 10 min. Images taken every 5 min from 20 min to 3 h. Images taken every 15 min from 3 h to 5 h.

**Figure 3:**
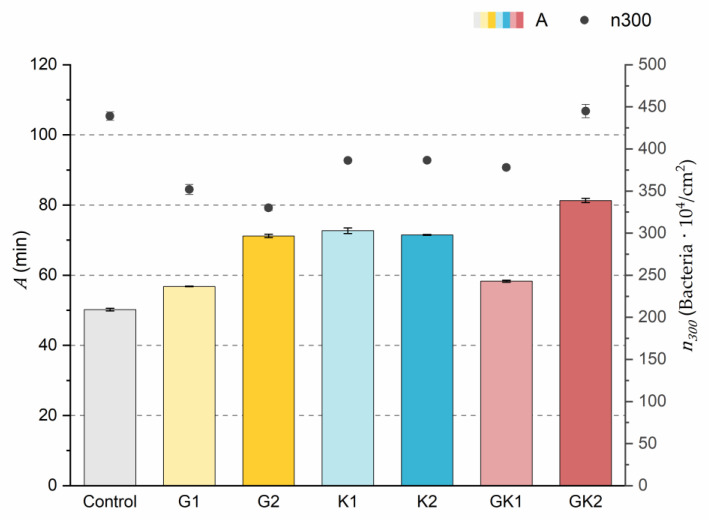
Parameters obtained from the exponential approximation of bacterial adhesion curves. A is the characteristic time of the system based on the exponential fit. $n_{300}$ is the bacterial coverage density corresponding to 300 min of adhesion. The left ordinate axis corresponds to the values of parameter *A*. The right ordinate axis corresponds to the data of parameter $n_{300}$.

Applying Equation (3) to the adhesion curves data for all conditions, we find that experimental results fit to an exponential increase with an R^2^ equal or really close to one. For example, in Figure 2, the curve fits with the experimental data in almost all the experimental time, with a slight deviation in the inflection zone just before reaching the stationary phase. Figure 3 shows the parameters previously defined for this first strategy.

The results indicated that the highest number of attached bacteria at the end of the experimental time ($n_{300}$) was reached in the control and GK2 conditions in the order of 440 × 10^4^ bacteria/cm^2^. In the rest of the conditions, approximately 20% fewer adhered bacteria were quantified, where the lowest value was found in the G2 environment (330 ± 6 × 10^4^ bacteria/cm^2^).

Analyzing in depth the characteristics of the adhesion systems, the parameter *A* is the time needed for the system to reach the final number of adhered bacteria if the initial speed were constant.

The values of the *A* parameter showed that the control medium was the one that caused the bacteria to reach the final bacterial density faster (50.2 ± 0.4 min) at a constant initial speed. In the case of systems with pathological concentrations such as GK2, we found a higher *A*, which means that this environment is the slowest. In analyzing the data, it is observed that the difference found between *A*_*G*2_ and *A*_*G*1_ was also evident between the two complex systems GK1 and GK2. Therefore, the longest time was obtained in the complex diabetic medium GK2, probably due to a synergy between G2 and K2. This fact would disfavor the initial bacterial colonization in the case of GK2, as increasing the characteristic time would slow down the bacterial coating of the surface.

#### 3.1.2. Second Analysis Strategy: Approximation by Sections

Through this approximation of the adhesion curves, we can extract similar information about the initial adhesion process. In the initial section, the systems exhibited a linear trend of adhesion during approximately the first 10 min, followed by an exponential rise that leads, at around 180 min, to a stationary phase in some systems and, in others, to the beginning of a new colonization stage.

**Figure 4:**
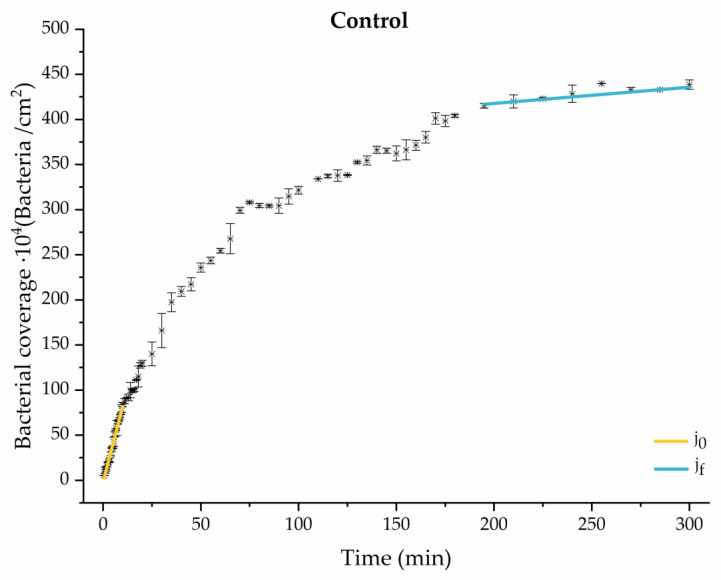
Approximation by sections of the evolution of bacterial adhesion on PLA (without diabetic enrichment in growth and adhesion). Images taken every 30 s during the first 10 min. Images taken every 60 s during next 10 min. Images taken every 5 min from 20 min to 3 h. Images taken every 15 min from 3 h to 5 h.

**Figure 5:**
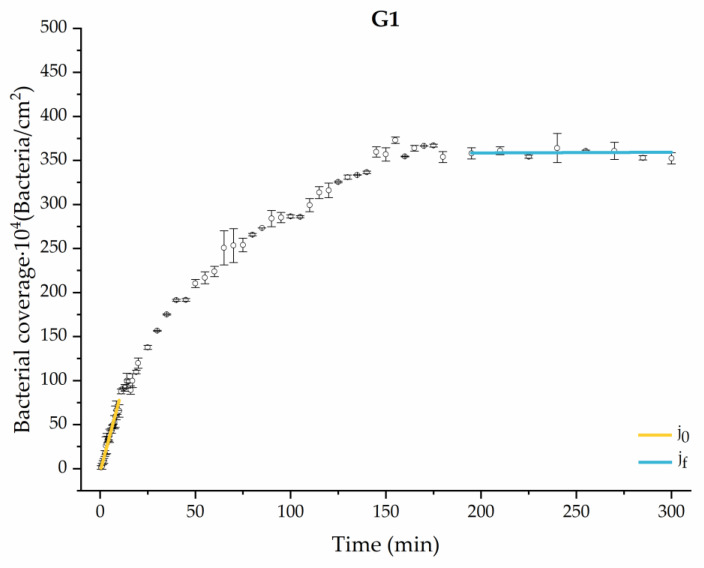
Approximation by sections of the evolution of bacterial adhesion on PLA when growth and adhesion are enriched with 0.9 g/L glucose (G1). Images taken every 30 s during the first 10 min. Images taken every 60 s during next 10 min. Images taken every 5 min from 20 min to 3 h. Images taken every 15 min from 3 h to 5 h.

**Figure 6:**
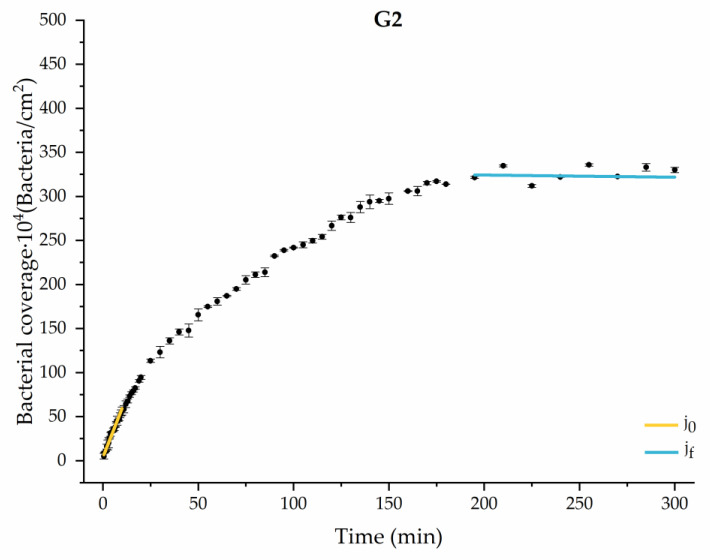
Approximation by sections of the evolution of bacterial adhesion on PLA when growth and adhesion are enriched with 1.9 g/L glucose (G2). Images taken every 30 s during the first 10 min. Images taken every 60 s during next 10 min. Images taken every 5 min from 20 min to 3 h. Images taken every 15 min from 3 h to 5 h.

**Figure 7:**
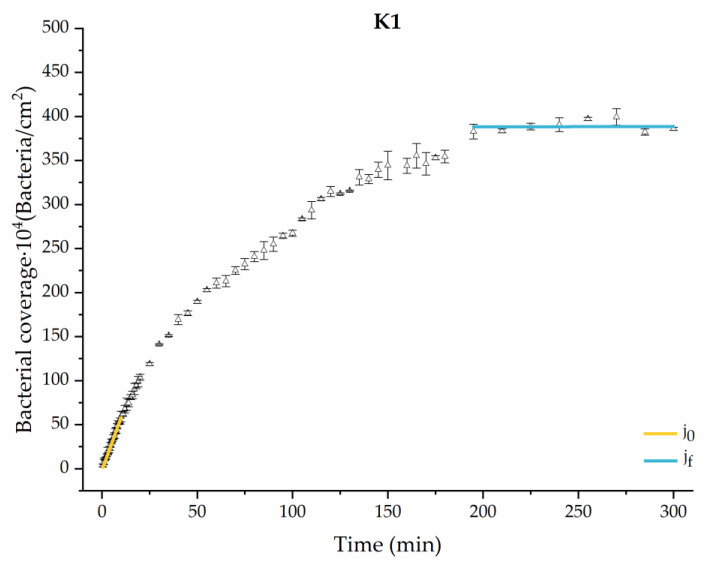
Approximation by sections of the evolution of bacterial adhesion on PLA when growth and adhesion are enriched with 1 mmol/L of ketone bodies (K1). Images taken every 30 s during the first 10 min. Images taken every 60 s during next 10 min. Images taken every 5 min from 20 min to 3 h. Images taken every 15 min from 3 h to 5 h.

**Figure 8:**
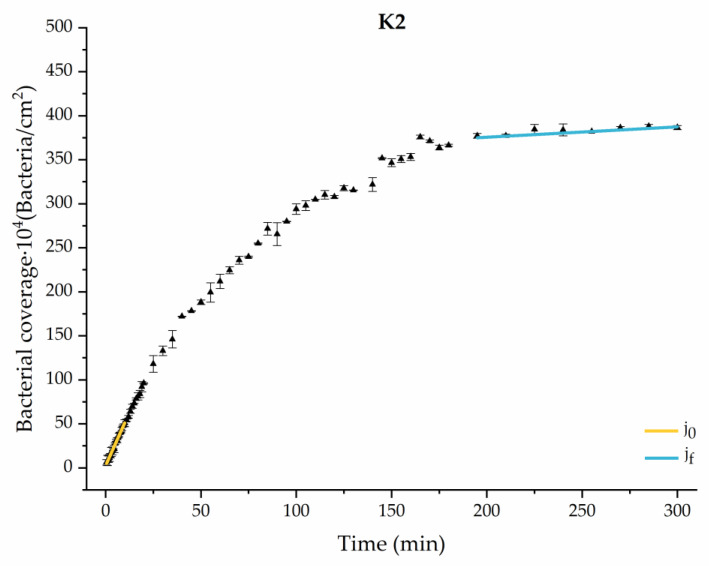
Approximation by sections of the evolution of bacterial adhesion on PLA when growth and adhesion are enriched with 9 mmol/L of ketone bodies (K2). Images taken every 30 s during the first 10 min. Images taken every 60 s during next 10 min. Images taken every 5 min from 20 min to 3 h. Images taken every 15 min from 3 h to 5 h.

**Figure 9:**
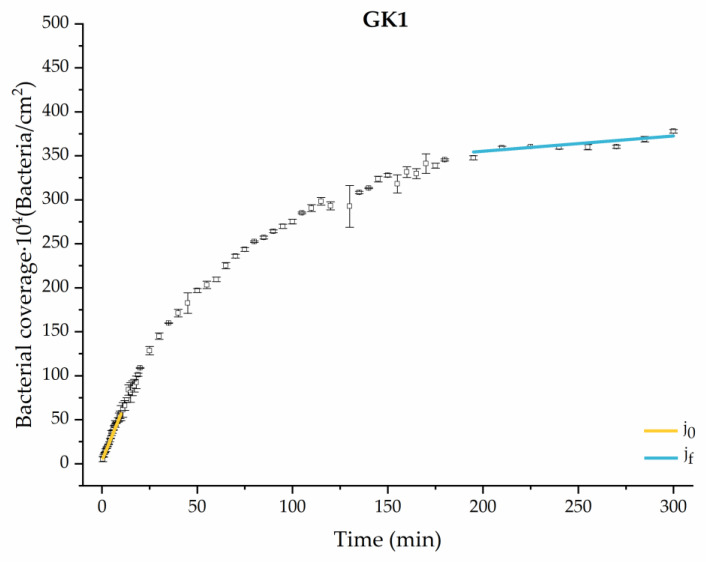
Approximation by sections of the evolution of bacterial adhesion on PLA when growth and adhesion are enriched with 0.9 g/L glucose and 1 mmol/L of ketone bodies (GK1). Images taken every 30 s during the first 10 min. Images taken every 60 s during next 10 min. Images taken every 5 min from 20 min to 3 h. Images taken every 15 min from 3 h to 5 h.

**Figure 10:**
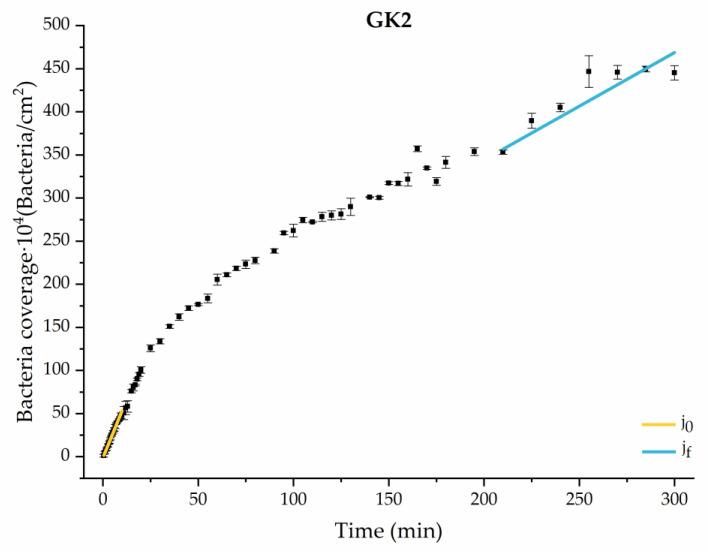
Approximation by sections of the evolution of bacterial adhesion on PLA when growth and adhesion are enriched with 1.9 g/L glucose and 9 mmol/L of ketone bodies (GK2). Images taken every 30 s during the first 10 min. Images taken every 60 s during next 10 min. Images taken every 5 min from 20 min to 3 h. Images taken every 15 min from 3 h to 5 h.

**Figure 11:**
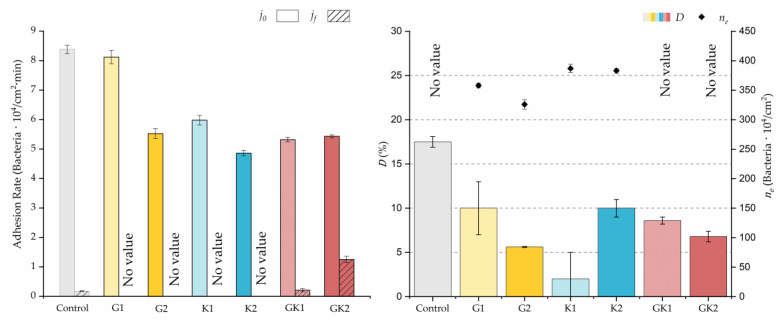
Parameters obtained from the approximation by sections of bacterial adhesion curves. $j_{0}$ is the initial adhesion rate during the first 10 min. $j_{f}$ is the adhesion rate at the end of the adhesion experiment. *D* is the percentage of reduction decrease. $n_{e}$ is the bacterial coverage density corresponding to the steady state of adhesion.

Figure 4, Figure 5, Figure 6, Figure 7, Figure 8, Figure 9 and Figure 10 show the curves of the number of attached bacteria as a function of attachment time for each of the systems studied. Additionally, the quantitative information obtained from these graphs is shown in Figure 11: plots in Figure 11a,b present the initial adhesion rates ($j_{0}$), the number of bacteria adhered in the steady state ($n_{e}$), in case this state is achieved, the new adhesion rate at the beginning of the new colonization stage, which corresponds to the end of the adhesion curve ($j_{f}$), if presented, and the reduction-decrease percentage (*D*) in the number of adhered bacteria after the passage of a liquid–air interface.

The data obtained for the initial adhesion rates agree with the *A* values in the case of the control system and for the system with non-pathological concentrations of glucose (G1). Although these systems show similarities during the first 180 min (Figure 2 and Figure 3), bacterial behavior in these two systems (C and G1) diverges in the stationary phase. Specifically, the control system did not reach a stationary number of bacteria, while G1 did ($n_{e}$ = 358 ± 4 × 10^4^ bacteria/cm^2^). In the hyperglycemic system (G2), the results (Figure 6 and Figure 11) show an initial adhesion time *(*$j_{0-G2}$ = 5.52 ± 0.17 × 10^4^ bacteria/cm^2^∙min) aligned with parameter *A*, and this initial linear trend of adhesion was longer than those for G1.

Among the systems enriched with the two components, glucose and ketone bodies, similar behaviors were observed in the last section of the adhesion process, where a second colonization process occurred after 180 min. Focussing on the GK2 condition, bacteria started the second colonization process prominently, exhibiting an adhesion rate of $j_{f-GK2}$ = 1.25 ± 0.11 × 10^4^ bacteria/cm^2^∙min, which was much higher than that observed for the control and for GK1. This fact could help to understand why in the pathological conditions associated with diabetes, it would be very difficult to eradicate any infection, since bacterial colonization would be far away from reaching a steady state with a “controlled” number of adherent bacteria. The rate $j_{f-GK2}$ may be responsible for the fact that $n_{300}$ of this system was the highest among all those studied ($n_{300}$ = 445 ± 8 × 10^4^ bacteria/cm^2^), despite being a system that starts with a low adhesion rate ($j_{f-GK2}$ = 5.53 ± 0.05 × 10^4^ bacteria/cm^2^∙min).

The effects of the liquid–air interface performed on the surfaces at the end of the adhesion processes yielded information as to which supplement has more/less capacity to promote the attachment of bacteria to the surface. There are authors who have indicated that, in addition to the surface properties of the bacteria and the substrate, this retention depends on the rate of passage of the interface [56]. Interestingly, the control system, without supplementation, showed the lowest attachment to the surface. The addition of any supplement increased bacterial retention, finding the highest attachment for bacteria within the K1 system. The remaining values were strongly conditioned by the amount and type of supplement used.

**Figure 12:**
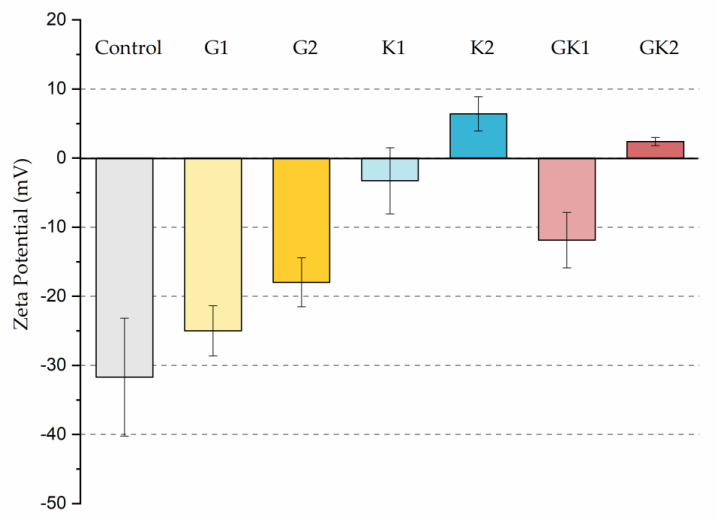
Zeta potential of the different systems analyzed in this work.

The different adhesive behaviors observed in the diabetic environments selected must not be caused by a single factor but must be the consequence of an interrelation of different mechanisms. As we have seen in our previous work in 2022 [36], bacteria grown in enriched media suffered a modification on their surface properties. In particular, the hydrophobicity and zeta potential are two properties closely related with the development of bacterial colonization. Our results showed an increase in relative hydrophobicity (G1 = 49 ± 5%, G2 = 47 ± 5%) for bacteria grown in the glucose-enriched media and, also, the absolute value of their negative zeta potential (G1 = −47 ± 2 mV, G2 = −49 ± 5 mV) with respect to bacteria grown in the control media (hydrophobicity 7 ± 6% and *ζ* potential −39 ± 2 mV). In systems enriched with ketone bodies, bacteria exhibit a much higher hydrophobicity (K1 = 75 ± 4% K2 = 77 ± 10%) and a more positive zeta potential in the case of K2 (K1 = −38 ± 2 mV, K2 = −15 ± 5 mV). In the combined systems, hydrophobicity remains high (GK1 = 72 ± 3% GK2 = 82 ± 3%) and zeta potential is also dominated by ketone bodies (GK1 = −39 ± 4 mV, GK2 = −22 ± 8 mV). This physical information of bacteria, previously published [36], has been complemented with similar information for the PLA surface. In the case of hydrophobicity, the water contact angle did not show variations, within experimental uncertainties, between the control sample and the diabetic supplemented samples, always displaying an average value close to 72 degrees [43]. However, the zeta potential of PLA suffered big changes after being in contact with diabetic supplements, as illustrated in Figure 12.

Interestingly, G1 behaves electrically as a control, within experimental deviations, while in the rest of the samples, the surface becomes less negatively charged, reaching positive values under high ketoacidosis conditions. These conditions (K2 or GK2) are the only ones that cause changes in both bacteria and PLA in the same sense: a decrease in the “natural” negative charge of the surfaces.

From a theoretical point of view, in aqueous environments, a higher bacterial hydrophobicity is associated with a higher adhesion to hydrophobic surfaces, as is the case of PLA, and a lower negative surface charge implies less electrical repulsion between electronegative surfaces. Although it is complicated to make a theoretical prediction of what may occur in each one of the supplementation environments, the changes in bacterial hydrophobicity, along with electrical changes in bacteria and PLA surfaces, can help to understand adhesive behaviors, especially in situations where changes are maximal.

In particular, only in the pathological case of GK2, a combined hyperglycemia and ketoacidosis environment, we found high hydrophobicity in bacteria (82 ± 3%), together with a reduction in the surface negative charge in both bacteria (−22 ± 8 mV) [36] and PLA (+2.4 ± 0.6 mV), which seem to be related to the promotion of the bacterial surface coating at long adhesion times.

The adhesive behavior of bacteria in complex systems is not easy to interpret, and this is widely known. For example, in Santore [57], it was suggested that bacteria immersed in a dynamic adhesion system with a mild shear might be more attracted to the presence of nutrients in the suspension than to the adhesion surface, due to the chemotactic gradient. Also, Boks et al. [58] showed how electrostatic repulsions in bacterial adhesion processes created energetic barriers that could be minimized through other mechanisms such as bacterial disposition-orientation [57,58]. These results could explain the behavior of bacteria in the system with pathological glucose concentrations, since lower adhesion rates and/or longer characteristic adhesion times, *A*, were observed in relation to these parameters in the enriched system with non-pathological glucose levels. In the case of the environments with ketone bodies, although the literature has little explored their effects on surface adhesion processes, different authors agree that these components do not create a comfortable environment for bacteria [36].

In particular, the decrease of 1.5 points in pH that occurs when supplementing an environment with ketone bodies [36] could alter the metabolic states and physiology of bacteria that would directly affect the bacterial adhesion strength on the surface [59,60]. Then, it is likely that this pH change is one of the reasons why bacteria subjected to ketoacidosis had a lower adhesion strength to PLA (*D* = 10 ± 1%).

Furthermore, while the surface hydrophobicity of the bacteria seems to be an important factor in bacterial retention to the surface, this can be altered by other surface properties like the electron-donating capacity. In the study by Begic et al. [61], no greater adhesion was observed in the more hydrophobic bacteria, because they possessed a major electron donor character as well as the substrate used, predicting a weak adhesion. Likewise, research has also found a correlation between a greater retention of bacteria on surfaces with an increase in ionic strength, justifying such changes with the variation of the zeta potential of the bacteria and the substrates. The work of Katsikogianni and Missirlis [62] focused on performing bacterial adhesion studies using a dynamic colonization model on glass functionalizing with methyl and amino groups self-assembled on alkylsilane monolayers. The colonization of two strains of *S. epidermidis* was studied in media with different ionic strength, concluding that increasing the ionic strength in the solution increased the adhesion, through the minimization of electrostatic repulsive interactions, as a consequence of the compression of the electrical double layers around the bacteria and the adhesion surface [62].

All these factors come into play in the environment of ketoacidosis and hyperglycemia (GK2), a system that promotes progressive colonization over time, suppressing the stationary adhesion phase and strengthening the attachment of bacteria to the surface.

### 3.2. Bacterial Metabolic State in the Adhesion Process in an Enriched Environment with Diabetic Conditions

In order to deepen the effect of diabetic environments on the behavior of bacteria during surface colonization, we studied the metabolic state of the bacteria at the beginning and at the end of the adhesion time.

**Figure 13:**
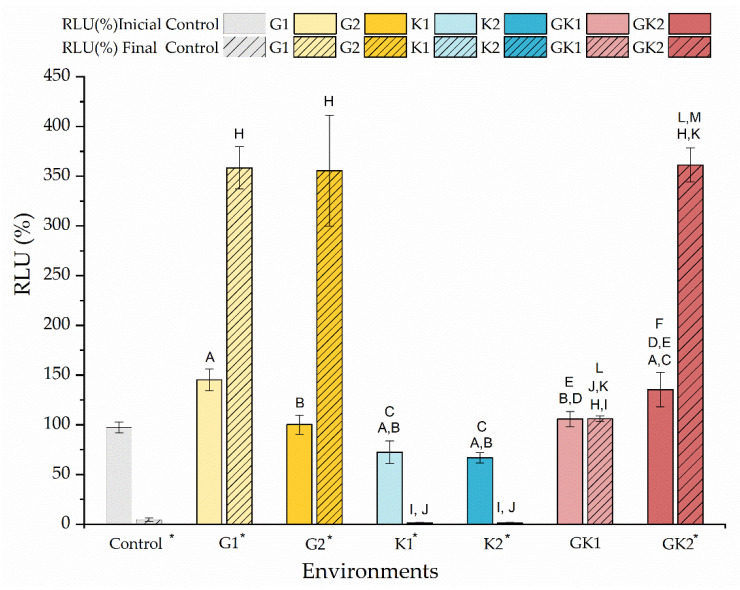
Percentage of the relative light units (RLU %) of the bacteria cultured in media enriched with glucose and/or ketone bodies, compared to the control samples before contact with PBS. The data are presented for samples before (no-striped columns) and after 300 min (striped columns) of contact with PBS with the same enrichment as culture media. Significant differences (*p* < 0.05) among samples before contact with PBS are marked as A, B, C, D, E, and F with respect to control samples G1, G2, K1, K2, and GK1, respectively. Significant differences (*p* < 0.05) among samples after 300 min contact with PBS are marked as H, I, J, K, L, and M with respect to control samples G1, G2, K1, K2, and GK1, respectively; control samples before contact with PBS have been taken as reference. Labels marked with * indicate significant difference (*p* < 0.05) between the samples after and before contact with enriched PBS, as indicated in the label.

Figure 13 shows the percentage of the relative light units (RLU) just at the moment of contacting the bacterial suspensions with the diabetic supplements in PBS and after 300 min of contact. The data are shown relative to the light units of the bacteria grown and suspended without enrichment, the so-called control, to which a value of 100% is associated.

The amount of ATP measured at the initial time indicated that there were not significant differences in the metabolic state of the bacteria exposed to different diabetic environments. Initially, the environments with non-pathological concentrations showed RLU values similar to the control; however, some differences can be detected between samples. In environments with pathological concentrations, bacteria under mixed diabetic environments had a higher metabolic activity than the control (GK2: 135 ± 17% RLU).

At the end of the adhesion time, that is, at 300 min, the results indicated the near absence of metabolic activity of bacteria in the control system and systems with ketone bodies. Instead, bacteria in the glucose system and in GK2, the complete diabetic system, showed an extraordinary increase in their metabolic activity, compared to their initial state, finding increments of 147% (G1), 255% (G2), and 167% (GK2). In the GK2 system, the increase in the metabolic activity of the bacteria over time, together with their favorable surface physical changes in relation to adhesion, may explain the dangerous adhesive inertia observed in the final stages of adhesion. Moreover, in this mixed system, one of the most significant features was the low percentage of detachment after the passage of the air–liquid interface (6.82 ± 0.55%). According to Pan et al. [63], a dense adhesion layer, such as that generated by GK2, would develop a compact structure that would help resist shear forces. Likely, in cases with marked metabolic activity, i.e., G1 and G2, the higher electrical repulsion shown by the bacterial surface against PLA was responsible for achieving moderate adhesive behavior in these two systems [36].

### 3.3. Biofilm Formation on Polylactic Acid Films in an Enriched Environment under Diabetic Conditions

In addition to the implications that bacterial metabolic changes may have on the dynamic adhesion processes in diabetic environments, it is also desirable to know how these bacteria may be able to develop biofilms on PLA surfaces. For this reason, biofilms were generated on the PLA surface for 24 h, with a culture medium enriched with the diabetic media already used.

**Figure 14:**
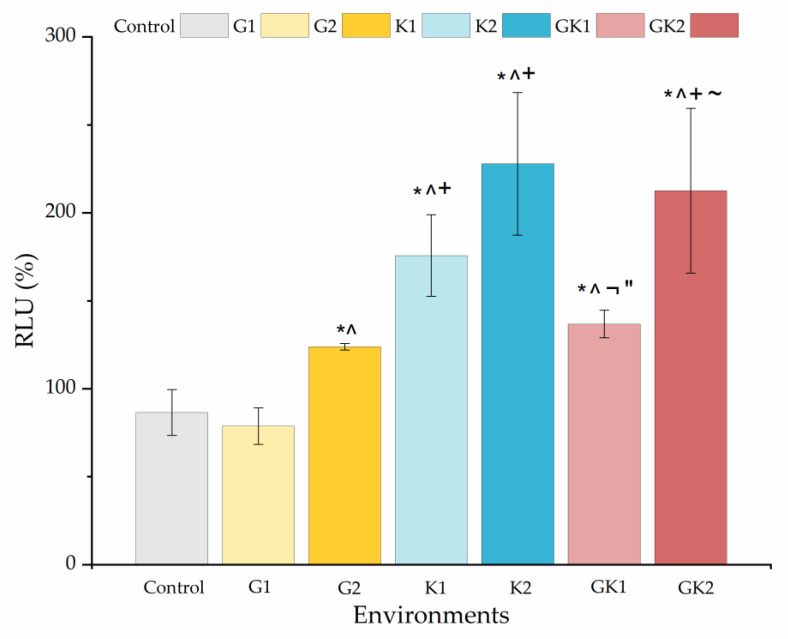
Percentage of the relative light units (RLU %) obtained from biofilms cultured in media enriched with glucose and/or ketone bodies, relative to the biofilm control growth in non-enriched media. Significant differences (*p* < 0.05) among samples are indicated by *, ^, +, ¬, ″, and ~ with respect to control samples, G1, G2, K1, K2, and GK1, respectively.

Figure 14 shows the percentage with respect to the control biofilm of the relative light units for all the systems studied. The presence of any diabetic enrichment favored biofilm formation. The case of ketone bodies deserves special attention, since they form the largest biofilm of all systems, especially in K2 (228 ± 41%). In the case of the mixed diabetic system GK2 (213 ± 47%), the ketoacidosis level seems to have been the dominant factor in the high biofilm formation.

In analyzing the changes obtained in the ATP quantification of bacterial metabolic activity and biofilm formation in diabetic supplemented environments, we can observe that biofilm formation is favored by ketone bodies, while in the planktonic state, it is glucose that triggers metabolic activity, being practically suppressed when ketone components are only present. Information on both must be complementary to understand what can happen in a real system, where planktonic bacteria are the ones that initially colonize a surface and, subsequently, these attached bacteria end up forming a biofilm.

The influence of ketone bodies on biofilm formation is little explored in the literature; however, it is documented that an acidic pH can enhance their development. This occurs because lower pH inhibits the production of extracellular proteases [64], promotes the binding of biofilm matrix proteins to cell surfaces [65,66], and stimulates the formation of microbial aggregates.

As for hyperglycemia, the literature reports that it enhances biofilm formation, as do other parameters such as alkalinity or osmotic pressure [67,68,69]. Specifically, the study by Lade et al. [69] analyzed the effect of different concentrations of glucose (0.5% and 1%) and NaCl added to TSB on the biofilm formation of different strains of methicillin-resistant (MRSA) and methicillin-sensitive *S. aureus* (MSSA). In addition, these authors performed genotypic and phenotypic characterization of *S. aureus* clinical isolates to determine their influence on biofilm-forming ability. Their results showed that glucose supplementation favored biofilm formation of all strains tested and that the formation rate depended on the strain used. In the study by Lee et al. [70], the formation of *S. aureus* biofilms on the surface of various materials was investigated by enriching TSB with 1% glucose [70]. These authors demonstrated how biofilm formation in hyperglycemic environments was largely dependent on substrate properties such as roughness, wettability, or hydrophobicity [70]. Also, the increase in biofilm formation caused by hyperglycemia may be associated with a decrease in the pH of the medium. In particular, the pH decreases in the medium in the GK2 system, associated with glucose metabolism, would favor biofilm formation [67,69]. Several investigations have revealed that biofilm formation is enhanced at pH 6, which is the one obtained in the GK2 culture media [67,71].

From a clinical point of view, the modification of bacteria in media with uncontrolled diabetic levels of hyperglycemia and ketoacidosis indicates the need for special monitoring of any implanted material made of PLA. The risk and virulence of biofilms that could form on any PLA surface in contact with the physiological media of these patients could double the risk compared to healthy patients. This result also aligns with the great importance of careful control of blood glucose and ketone bodies for those with diabetes. Certainly, this issue points the way for further research. The altered behavior of bacteria, when present in an uncontrolled diabetic environment, is far from our control, but, considering the benefits of bioresorbable PLA, the search for PLA enriched with compounds that can prevent adhesion and/or biofilm growth of bacteria under these conditions is in the focus of our future research.

## 4. Conclusions

The data shown in this research indicate that the colonization of *S. aureus* on PLA surfaces in the presence of diabetic components such as glucose and/or ketone bodies is not associated with a single factor but is a process in which the properties of the surface of the bacteria, the substrate, and the medium in which the colonization process takes place come into play.

Ketoacidosis environments always reduce the net negative charge of bacteria and the host–PLA surface, thereby decreasing the “natural” repulsive forces in aqueous environments.

In the presence of pathological concentrations of glucose and ketones (GK2), the results of the different assays point to an increased pathogenicity of the bacteria.

Far from achieving a stationary state of adhesion, the GK2 system activates a new adhesive process at that point, motivated by a very active metabolic state of the bacteria at this time.

Moreover, after 24 h, GK2 triggers biofilm formation, based on ATP quantification, by about 200% relative to a system without diabetic enrichments.

This research highlights the particular importance of the need for good control of diabetic patients, specifically if they are using any implanted device made of PLA, as the risk of biofilm growth can double in uncontrolled patients compared to those with normal levels of glucose and ketone bodies.

## Data Availability

The data presented in this study are openly available in Dehesa at https://dehesa.unex.es/ (6 July 2024).
